# Annotations

**Published:** 1899-02-25

**Authors:** 


### ANNOTATIONS.

### The Feeding of Prisoners.

THE report of the Departmental Committee on Prison Dietaries has just been issued, and its recommendations, we need hardly say, have an upward tendency. The two diets which have led to the greater number of protests are the Class I. and Class II. in the local prison diets; and the committee, bearing in mind their instructions to the effect that "the ordinary prison diet is not to be regarded as an instrument of punishment," have found no difficulty in recommending that both of them shall be abolished, and that new diets of improved nutritive value and greater variety shall take their places. It seems probable that the increased variety and palatableness which will thus be introduced will really be of more importance than their improved nutritive value. How misleading are mere chemical estimates of food values is shown by the fact that, notwithstanding the meagreness of certain prison diets, a considerable proportion of what is called "stirabout" is habitually left unconsumed by the prisoners. Now, we are not particularly squeamish about the treatment of law-breakers, and we see nothing specially sacred about a man's stomach which would make us hesitate to use it as a vehicle of punishment, were it not for the fact that if a man's strength is reduced in prison he is rendered unfit to earn his living by honest labour on his discharge, and is thus driven back into crime again. Unless then we are prepared to cast on one side all idea of rendering prison life reformatory, and of enabling a man on his discharge to make a fresh start, we must turn him out fit and strong, and this puts entirely out of the question all idea of punishing prisoners, especially those under short sentences, by lowering their diet. The changes recommended may possibly have the effect of rendering imprisonment somewhat less deterrent than has been the case to some of the rougher element; and this is much to be regretted. On the other hand, it is to be hoped that it will result in prisoners being less liable on their discharge to yield to the temptation to revert immediately to crime. We are reminded of the well-known story of the painter who, when asked what he mixed his colours with, answered, "With brains, sir, with brains"; and it would appear that the members of this committee, in arranging the prisoners' food, have not been governed entirely by chemical theories of nutritive value, but have mixed their diets "with humanity."

### An Antitoxin Story.

THE *Practitioner* of this month gives another of the admirable short stories, with which it endeavours to lighten the dulness of medical literature—this time dealing with the antivivisectionists and antitoxin. The tale opens with three doctors talking in the club window. One of them was greatly upset. Some time previously he had attended the child of Sir Rupert Uffington, who lay at death's door with diphtheria. He had strongly urged antitoxin, pointing out that the child must die if it were not used, yet the father, being a leading antivivisectionist, had refused it; and now came a paragraph in the paper, saying that, not only had the child recovered, but that the father had been making capital of the case at a public meeting. After much talk the irate doctor ends by saying, "Don't think I'm not glad the little beggar lived. But it's no thanks to his parents." "The old doctor smiled at him mysteriously—I could see it even in the dusk—dropped his cigarette end into the fire, and came and sat down leisurely between us. 'I remember a very similar case, if it won't bore you, eh?' he began. 'One evening an old patient of mine, a beautiful creature whom I had watched grow up from the nursery, until I lost sight of her on her marriage, came to me in an agony of distress. Her child, too, seemed likely to die of diphtheria, and her husband stubbornly refused to let the doctors make use of the new treatment; but she was determined, if she could, to administer it herself without his knowledge. It would be difficult, she admitted, for the child was jealously isolated, and she was scarcely allowed in the room. But that night she thought she could contrive to have it for an hour to herself. Wouldn't I help her as an old, old friend? Of course I demurred and raised a heap of difficulties. I showed her the syringe, fully expecting to scare her from her project. She blenched when she saw the needle, and felt its point at her skin, as I demonstrated its use on the back of her hand. But yet she insisted. I had forgotten what a brave little heart she had always shown even as a child behind her nervous, uncertain manner. Well, to cut a long story short, in the end she went off with syringe and serum, a supply of which I luckily happened to have by me, and I learnt afterwards that that poor, shrinking, inexperienced girl had dug the needle twice into her own flesh before she would trust her skill to use it.' 'What a ripper!' I cried. 'And she saved her child?' 'Her child recovered,' said Dr. Parridine, with scientific caution. 'I suppose it was all very wrong and unprofessional on my part, but I am a soft-hearted old buffer, and if the case recurred, hang me if I wouldn't do it again.' 'Ah!' sighed Bredon, 'if Lady Uffington had shown but half her pluck!' 'My good Bredon,' said the old doctor very deliberately, smacking his lips, as it were, over the effect which he had all along been preparing, 'it—it was Lady Uffington.' 'Great heavens!' cried Bredon, after a moment of dumfounded silence. 'You don't say so! Lady Uffington! Hurrah! Why then, Sir Richard and I were not so wrong after all.'"

### "Not at Home."

THE work of carrying out the provisions of the Vaccination Act will probably be by no means so simple as it appears on paper, and, among the various difficulties which may be expected to crop up, not the least irksome will probably be that arising from the parents being "Not at home" when the vaccinator calls to offer vaccination, notwithstanding that he has sent proper notice of his intended visit. How far this nuisance may interfere with vaccination work may be judged by the fact, reported from Liverpool, that on a certain date a public vaccinator notified fifteen families that he would call to vaccinate the children on a certain day, but on calling on the appointed day he found only three of them at home. This is bad enough in a town; but in the country, where long journeys have to be made, such action on the part of the parents would be enough to drive an unfortunate public vaccinator to despair.

